# Health provider perspectives of Village Health Team-delivered oral HIV self-testing among men in Central Uganda: a qualitative evaluation using RE-AIM framework

**DOI:** 10.21203/rs.3.rs-3816613/v1

**Published:** 2024-01-26

**Authors:** JOANITA NANGENDO, Rhoda K. Wanyenze, Gloria O. Obeng-Amoako, Mercy Muwema, John Mukisa, Jaffer Okiring, Jane Kabami, Charles A. Karamagi, Fred C. Semitala, Joan N. Kalyango, Moses R. Kamya, Anne R. Katahoire

**Affiliations:** Makerere University School of Medicine; Makerere University College of Health Sciences; Makerere University College of Health Sciences; Makerere University College of Health Sciences; Makerere University College of Health Sciences; Infectious Diseases Research Collaboration; Infectious Diseases Research Collaboration; Makerere University College of Health Sciences; Makerere University College of Health Sciences; Makerere University College of Health Sciences; Infectious Diseases Research Collaboration; Makerere University College of Health Sciences

**Keywords:** perspectives, VHT-delivered, oral-HIV self-testing, VHTs, facility-based-health-workers, Uganda

## Abstract

**Background:**

HIV self-testing (HIVST) is a practical and effective way to provide HIV testing services to at-risk and underserved populations, particularly men. Utilizing Village Health Teams (VHTs) could enhance community-based delivery of oral HIVST to reach the last un-tested individuals who may be at-risk of infection. However, little is known about what VHTs and facility-based healthcare workers think about facilitating oral HIVST and delivery of subsequent HIV services. We investigated the views of health providers on oral HIVST delivered by VHTs among men in rural communities in Central Uganda.

**Methods:**

We conducted a qualitative study in Mpigi district, interviewing 27 health providers who facilitated oral HIV self-testing among men. The providers consisting of 15 VHTs and 12 facility-based health workers were purposively selected. All interviews were audio-recorded, transcribed verbatim, and translated to English for a hybrid inductive-deductive thematic analysis. We used the Reach, Effectiveness, Adoption, Implementation, and Maintenance (RE-AIM) Implementation Science framework to generate and categorize open codes.

**Results:**

In terms of reaching men with HIV testing services, the providers considered HIVST to be a fast and convenient method, which could boost HIV testing. However, they also had concerns about its accuracy. In terms of effectiveness, HIVST was perceived as a reliable, user-friendly, and efficient approach to HIV testing. However, it depended on the user's preference for testing algorithms. Regarding adoption, HIVST was considered to enhance autonomy, well-suited for use in the community, and offered opportunities for linkage and re-linkage into care. However, at times HIVST faced hesitance. As for Implementation, VHTs had various support roles in HIVST but had concerns about social insecurities and delays in seeking subsequent facility-based services after HIVST. Regarding Maintenance, providers recommended several ways to improve oral HIVST including; optimizing tracking of HIVST distribution and use, improving linkage and retention in care after HIVST, diversifying HIVST for combined HIV prevention packages and including more languages, broadening sensitization among potential HIVST users and health providers, differentiating distribution models, and prioritizing targeted HIVST efforts.

**Conclusion:**

HIVST has the potential to increase testing rates and engagement of men in HIV services. However, for it to be implemented on a population-wide scale, continuous sensitization of potential users and health providers is necessary, along with streamlined structures for tracking kit distribution, use, and reporting of results. Further implementation research may be necessary to optimize the role of health providers in facilitating HIVST.

## Background

HIV self-testing (HIVST) is globally recommended as an effective and innovative approach for extending HIV testing services to the last untested persons [1]. HIVST has several competitive advantages, including being painless, quick, easy-to-use, and convenient for use in clinical and non-clinical settings (1). In Sub-Saharan Africa (SSA), HIVST has been widely researched among various priority populations, including men in the general population [2], men who have sex with men [3], male partners and couples [4], fisher folk [5], as well as studies assessing the feasibility and effectiveness of distribution strategies [6, 7]. Other research explored factors influencing the uptake of HIVST [8, 9], and linkage to care after HIVST [10-16], and have cumulatively contributed evidence to improve utilization of HIVST among at-risk sub-populations in resource-limited settings. In Uganda, men have been identified as a priority population at substantial risk of HIV infection; however, they often fall behind in accessing HIV care [17]. Several barriers to HIV testing among men in Uganda and SSA have been documented [18-23], and in-part explain why they are often missed in population-wide HIV services [20, 24]. These barriers include; masculinity norms, fear and stigma related to testing HIV positive [23], high mobility, unavailability related to-work, leisure and social engagements [25], and discomfort of seeking care in a female-oriented healthcare system [26, 27].

In our previous study of men in the general population in Uganda, we found that providing oral HIVST through village health teams (VHTs) increased testing rates, disclosure of HIV status, and use of HIV services at healthcare facilities. This included confirmatory testing and the start of antiretroviral therapy for individuals who received a positive HIV diagnosis [9, 11, 23]. In addition to the attributes of independence and individual autonomy experienced in using HIVST, men still relied on VHTs to interpret the self-test instructions and results, and to seek guidance on confirmatory testing and additional HIV services [9, 11]. It is still unclear how VHTs and facility-based healthcare workers who support oral HIVST feel about being involved in the delivery of such services. Provision of health services depends on various factors, including interactions between health policies, healthcare delivery systems, at-risk populations, healthcare service utilization, and consumer satisfaction [28]. Clear understanding of user experiences and health provider perceptions of a service is crucial to ensure consumer satisfaction. In the context of HIV Self-Testing (HIVST), this study aimed to explore the perspectives of facility-based health workers and VHTs who supported oral HIVST among men in Mpigi district, Central Uganda.

## Methods

### Study design and setting

Between October 2018 and June 2019, a single prospective cohort study was conducted to evaluate oral HIVST among men in 30 villages of Mpigi district in Central Uganda. Following this study, an explanatory qualitative study was conducted from June to August 2019. Briefly, the study team, led by VHTs, sensitized sampled communities about oral HIVST and recruited participants. Each participant was given an oral HIVST kit and a leaflet containing information on how to access HIV confirmatory services within the study district. Ten days after 10 days distributing the kits, the study team checked if the participants had used the HIVST kit. At one month after distribution, the team followed up to see if the participants had accessed facility-based HIV confirmatory testing and disclosed their HIV status. At three months, the team checked if participants who tested positive for HIV had initiated antiretroviral therapy (ART) [9, 11].

Mpigi District is situated in Central Uganda, with its headquarters located approximately 37 kilometres towards the west of the national capital city, Kampala. The district is divided into seven sub-counties, 56 parishes, and 339 villages, with a predominantly rural population of over 286,600 people, of which 50% are male and 83% reside in rural areas [29]. About 80% of the population is literate, 59.2% are involved in subsistence farming, and a smaller proportion engage in small-scale businesses such as fishing, retail shops, and transportation, particularly as boda-boda [commercial motor-cyclist] and taxi drivers [29]. The district has a total of 41 health facilities. Out of these, 21 are owned by the government and offer HIV testing and ART services. The remaining facilities are owned by private not-for-profit (PNFP) and private for-profit (PFP) schemes, with 11 and 9 facilities respectively [30]. The health facilities are supported by a network of over 2500 VHTs to improve equity and access to health services in communities [30-32]. VHTs provide various health services including; health education on maternal and child care, HIV/AIDS, TB, reproductive health, immunization, nutrition, and sanitation [31, 32]. Despite national efforts, Mpigi district's HIV prevalence is 8.1%, far above the 5.8% national average [17].

### Study participants, sampling and sample size estimation

Facility-based health workers at designated sites offered HIV testing and ART services in Mpigi district, while VHTs facilitated oral HIVST in the selected study villages. All participants were purposively selected based on their willingness to share their experiences and availability to interact with the study team. The optimal sample size was determined based on data saturation [33], when no new information was obtained from all new data that was collected.

### Data collection

We conducted 27 in-depth audio-recorded interviews, with 12 VHTs and 15 health workers based in facilities. Each interview lasted approximately 45 minutes. The interviews were conducted by research assistants (RAs) who had experience in conducting qualitative research and held a minimum qualification of a diploma in social work. They were fluent in both English and Luganda, the commonly spoken local language. The interviews were conducted at a convenient location and time for each respondent. A pre-tested guide with open-ended questions tailored to suit the caliber of the participant was used during the interviews. The RAs used separate interview guides for the VHTs and facility-based health workers. At the beginning of each interview, the participants were asked for permission to record the conversation for future analysis. They were also asked to give written consent to join the study. They were reassured that all the information collected would be kept confidential and would be fully de-identified before the analysis and dissemination of the study's findings. The original interview audios were backed up and stored on a private, password-protected folder on the principal investigator's (JN) computer.

### Data analysis

All interview audio recordings were transcribed verbatim by two independent RAs and then translated to English for analysis. Initially, JN checked the English transcripts for accuracy and completeness by concurrently reading through each transcript and listening to the respective interview audio. Our analysis followed a hybrid inductive-deductive thematic approach [34], consisting of six steps: familiarization, coding, generation, review, definition and naming, and theme writing from data [35]. JN created the first set of open codes, categorized them, and established themes. After that, JN shared the initial codebook with two colleagues (JK and MM) for independent review. The revised codebook was then shared with a senior qualitative researcher (ARK) for final consensus. We incorporated the feedback given by ARK to finalize the categorization of the codes, resultant themes, and the respective illustrative quotes.

This being an implementation science study, we employed the Reach, Effectiveness, Adoption, Implementation, and Maintenance (RE-AIM) framework domains in categorizing the codes and themes derived from the data [36-38]. We defined the domains of RE-AIM framework as stated by Holtrop et al in 2018 [36]. In relation to our study, the RE-AIM domains were defined as follows: 1) Reach referred to all expressions of why men accepted or declined participation [use of VHT-delivered oral HIVST] and descriptive characteristics of participants versus non-participants that were not available from prior quantitative data or records; 2) Effectiveness referred to key qualitative issues relevant to understanding whether various stakeholders found the effectiveness findings meaningful, why the intervention [VHT-delivered oral HIVST] produced different patterns of results across different RE-AIM dimensions, reasons for differences in results across subgroups and why unanticipated negative results were observed [36]; 3) Adoption referred to the key qualitative issues parallel to those of reach, but at levels of settings and staff/implementers such as understanding why different organizations - and staff members within the organizations - chose to participate or not; and understanding the complex or subtle differences in those organizations and staff members in terms of underlying dynamics and processes [36]; 4) Implementation as understanding the conditions under which consistency and inconsistency occurred across staff, setting, time, and different components of program or policy delivery [36]; and 5) Maintenance as understanding program sustainability and the reasons why individual benefits continued or faded, and why the organization delivering the intervention decides to continue or discontinue the intervention – which are important for future program design and scale up [36]. Ultimately, we presented themes under domains of the RE-AIM framework.

### Trustworthiness of the study data

We utilized a number of approaches to ensure trustworthiness of our study data. First, we followed the Consolidated Criteria for Reporting Qualitative Research (COREQ) guidelines [39], and used the 32 item COREQ checklist to ensure consistency and transparency in the collection, management and analysis of the study data. Second, reflexivity. Since the parent study involved only cis-male gender participants, who were supported by predominantly cis-male gender health workers, even the interviews reported in this study were conducted by cis-male gender research assistants (RAs) to ensure credibility and conformability of the research findings [40]. Third, prolonged engagement, the RAs who conducted the interviews in this study had prior involvement in the parent study and had interacted with the study participants, VHTs and facility-based health workers for over a month in the study communities so found it easy to establish rapport needed to conduct the interviews [40]. Fourth, during analysis, the final codes and themes reported in the study were preceded first by independent review of study team members and discrepancies resolved by group consensus following review all the independent reports [35].

## Results

Perspectives of facilitating VHT-delivered oral HIVST among facility-based health workers and VHTs included; HIVST included being a quick and convenient way to reach men with HIV testing services, a boost for health care seeking, a streamlined and trustworthy approach of HIV testing, ability to enhance autonomy about one’s health, well-adapted for community work, and offering opportunity for linkage and re-linkage into care. On the downside, there were concerns about accuracy, hesitance to use HIVST, social insecurities, and delays in accessing subsequent HIV services after HIVST. There were also recommendations to optimize tracking of HIVST distribution and use, augment linkage and retention in care after HIVST, diversify HIVST for HIV prevention services, broaden sensitization of HIVST users and health providers, and differentiate HIVST kits distribution to suit varied priority populations. The perspectives were presented using the domains of the RE-AIM framework as detailed below.

## Reach

This domain referred to expressions of why people accepted or declined participation and description characteristics of participants versus non-participants – according to the providers which individuals embraced oral HIVST and who were those who did not and why [36].

Health providers perceived HIVST as time-saving and as convenient for **reaching men with HIV testing services**. HIVST was perceived to be painless, accessible, and as ensuring users’ privacy.

“… it is going to bring services nearer to people because most people find difficulties to come to the hospital to test themselves.”

(Health worker, Bunjako Health Centre III)

“…there are people who fear going for testing when there are many others around. They fear that there will not be discretion in the sense that other people may see them coming out with a gloomy face and conclude that they are sick. I knew this method is going to be good being that one can test themselves from their homes and act accordingly…”

(VHT Bukasa - Farming community)

Health providers shared that HIVST could **boost health care seeking** especially among the hard-to-reach sub-groups including men and key populations. They said HIVST boosted clients’ confidence to test including first-time testing, consideration to seek confirmatory testing, encouraging repeat or regular testing even among partners, and prompting users to embrace HIV prevention or initiate ART early.

“You may even find that he has the virus but when he did not know, but because of that morale that they [VHTs]give to a person [from HIVST] it is what has pushed him to come.”

(Health worker, Mpigi Health Centre IV)

“…a person leaves their home after HIVST let’s say he is positive, they [the VHT] will have already told him something about the advantages of starting treatment and will know his life.”

(Health worker, Ggoli Health Centre III)

However, health providers worried that users who obtained negative HIVST could be discouraged from seeking confirmatory testing yet there could be possibilities of false results in case of unsupervised HIVST.

“…these people who test themselves think that it is done even if he does not go ahead [seek confirmatory testing]. But our HIV guidelines say that a person has to be tested…two times to be confirmed, but these ones if they test themselves and find that they do not have [HIV], they disappear and they do not even come back to the hospital…”

(Health worker, Mpigi Health Centre IV)

The providers also revealed varied concerns about the **accuracy of oral HIVST**. Some had believed HIVST was accurate so users were contented with the results they obtained and could be used to affirm one’s sero-status. On the contrary, others had concerns on the potential of false results which could raise confusion unless users obtained confirmatory testing.

“Many people were pleased with it as it revealed correct results. We used it ourselves before later testifying to the people.”

(VHT Nabusanke – Trading Centre)

“…a person can test himself and he does not pass through the right procedures and thinks that he does not have HIV yet he has and he ends up becoming a problem…”

(Health worker, Mpigi Health Centre IV)

## Effectiveness

This domain focused on key qualitative issues relevant to understanding whether various stakeholders (VHTs and facility-based health workers) found the effectiveness findings meaningful, why VHT-delivered oral HIVST (the intervention) produced different patterns of results across different RE-AIM dimensions, reasons for differences in results across subgroups and why unanticipated negative results were observed [36].

Health providers observed that HIVST was **a streamlined approach that could be trusted for HIV testing**. They felt that it eased the process of sero-status confirmation, with users able to confirm their HIV status after testing more than once; first using oral HIVST then followed by traditional blood-based tests in the national HIV testing algorithm. This could prompt them to initiate ART where their HIV test results were positive and to initiate preventive measures if their test results are negative.

“… it is true that saliva can test HIV but that patient was taken through all steps that are required for HIV testing. They had got most of that information, but we explained to him what he wanted to do…”

(Health worker, Ggoli Health Centre III)

“If someone knows his status as early as possible, he can protect himself in anyway at an early age. If I find out now when I have used that kit that I am sick [HIV positive], then I should go for that service as early as possible.”

(Health worker, St. Monica health Centre III)

Providers observed that HIVST was **user-friendly** and VHTs understood the process very well and could guide users whenever they were approached. They also reported that during confirmatory testing, HIVST users informed them that the test kit was easy to use and interpret, and that encouraged many men to test. However, providers felt that HIVST was prone to error hence may need to be used under supervision especially among lay users to minimize user errors.

“The advantages I saw…is that it [HIVST kit] is friendly to a person who uses it whereby it does not mislead….”

(Health worker, Bunjako Health Centre III)

There were guidelines to follow during testing for instance; It is necessary to wait for fifteen to twenty minutes after eating or brushing your teeth before testing…”

(Male, VHT Bukasa – Farming community)

“Some of them tested themselves after eating. Sometimes it does not come out very well [could be false HIVST results].”

(Health worker, Mpigi Health Centre IV)

According to providers, the preference for HIVST algorithms was dependent on the user. They observed that the majority of people still favoured blood-based HIV testing as the standard, while some opted for supervised oral HIV testing. Providers also expressed concerns regarding the accuracy of oral HIV testing, which may cause interruptions to blood-based testing.

“Some welcomed it [oral HIVST] and others didn’t as they accepted only the blood test method.

(VHT Musa)

“I think that for it [oral HIVST] being a new method. They do not believe in it saying that they are lying to them it cannot be correct.”

(Health worker, Nkozi Hospital)

“I fear that those who have never used the blood tests at the hospitals and only used this method [oral HIVST] may compromise to never opt for the former…”

(VHT Nkozi – Trading Centre)

## Adoption

This domain focused on key qualitative issues parallel to those of reach, but at levels of settings and staff/implementers such as understanding why different organizations - and staff members within the organizations - chose to participate or not; and understanding the complex or subtle differences in those organizations and staff members in terms of underlying dynamics and processes [36].

Health providers stated that HIVST provided individuals with greater control over their own health. They felt that HIVST promotes positive health-seeking behaviors, such as a willingness to undergo confirmatory testing, disclosure of one's sero-status, and notifying partners of potential risks.

“They [people who had self-tested] also came and we confirmed to them…when somebody tested himself and was negative, when he came here [to the health facility], he was also negative. When we got one who was positive from the other side when he came here he was also positive.”

(Health worker, Kituntu Health Centre III)

“…the doctors will ask me that Mr. J does your wife know your life status and you say no. …I just request that you come with her to the hospital or I can call you at home and we talk with her slowly.”

(Health worker, Nswanjere Health Centre III)

Additionally, providers observed that counseling services offered during HIVST could help individuals overcome anxiety related to confirmatory testing, which in turn could lead to greater acceptance of test results and a greater desire to disclose one's sero-status.

“… by the time a person leaves their home let’s say he is positive, they had already told him something such as the advantages of starting medicine and knowing his life status. So most came from that side to start medicine because they already came with what you had told them.”

(Health worker, St. Monica health Centre III)

According to the VHTs, HIVST is suitable for community work and can be used in clinical and non-clinical settings, including workplaces, to reach those who have not yet been tested. They found that HIVST integrated well with their other community activities, making it easy to mobilize and educate men about the testing process. Additionally, HIVST reduced their workload as men could conduct the test independently with minimal assistance.

“I encouraged and convinced them since we met them at their homes, places of work and some in the gardens…I spoke at different functions including funerals which really helped me to gather people.”

(VHT Luwala – Farming community)

“…they came with some information. For us we just added on and they accepted their status and started treatment for those who were positive.”

(Health worker, St. Monica health Centre III)

Health providers saw HIVST as a positive opportunity to connect first-time testers who were diagnosed HIV-positive with care. It also provided an opportunity to re-link clients who were previously diagnosed with HIV, but may have discontinued care and needed a favourable entry point to resume care.

“…you make sure that you give the person good information… the only thing when he has turned positive is going into care but you have to convince this person. To accept that yes I have to go and start having that chronic care.”

(Health worker, Kituntu Health Centre III)

“… you give him freedom and a chance to decide where he is going to take the medicine [ART] from maybe he has decided to take it from here and we start from here with those who had started and later left it [ART].”

(Health worker, Bunjako Health Centre III)

According to health providers, certain individuals remain hesitant to utilize HIVST, despite its convenient nature. This may be due to apprehension surrounding the potential to receive a negative result, particularly if a positive HIV diagnosis is confirmed. Concerns regarding self-harm, unresponsiveness, and coercion also exist, which could deter individuals from seeking the necessary assistance, particularly if HIVST is conducted without supervision.

“… the problem I see is that you can test him [with a self-test kit] and he runs away or he does something wrong after testing him because he won’t have a counselor when he is testing…he may not first sit down to think what to do.”

(Health worker, Nkozi Hospital)

… if a person has more money and energy than you. For him what he decides that is it….it becomes easy for them to manipulate you… and he tells you that put here your saliva even if you don’t want.

## (Health worker, Nindye Health Centre III)

### Implementation

This domain focused on understanding the conditions under which consistency and inconsistency occurred across staff, setting, time, and different components of program or policy delivery [36].

VHTs had several support roles in HIVST. These included general sensitization on HIVST, pre- and post-test counselling and social support to users of HIVST. They also followed up with those who tested positive for HIV, and helped them access confirmatory testing, prevention and care services. In addition, they assisted with sero-status disclosure and partner notification.

“… we have to thoroughly educate the people on what to do and then counsel them. Secondly, we have to ask them to be open and trust the VHTs so that I can help them throughout the process…”

(VHT Bwanya - Farming community)

“They got help from the VHT. They would ask according to their results and whether positive or negative, then would proceed to the health center or hospital to confirm using the blood test method.”

(VHT Ggolo – landing site)

“…even when they [doctors] come and test, they don’t follow-up which I [VHT] proudly do when I know that one of my people is positive… we know the people better and their whereabouts which makes follow-ups easier.”

(VHT Bwanya – Farming community)

Unfortunately, there were occasions when potential users resisted HIVST, which impacted other activities like counselling, especially for those who were on the move.

“…I [VHT] also did the same among the people telling them that this method helps you to self-test and know your status… It was not an easy task as I could only convince six out of ten people… I used tactics I knew from my experience… some people came to test and later witnessed for others.”

(VHT Musa - Island)

Providers expressed concern about the possibility of **delays in seeking subsequent facility-based services** after HIVST. They said facilities often have long waiting periods before one can be tested, and the distance and transport costs may also discourage HIVST users from accessing and utilizing HIV services provided at the health facility.

“The challenge that they [those who had self-tested] found that sometimes they found here patients who had come to treat other diseases so they had to wait a bit while we worked on others and afterwards they entered to be worked upon.”

(Laboratory Technician, Mitala Maria Health Centre III)

“I encouraged them to go to hospital to get free medicine. Maybe one may bring up an issue of transport to the hospital which I can’t help with.”

(VHT Nabusanke)

“The hospitals are too far and it is difficult for people to go there. For instance, there is a man that came after visiting the hospital all the way to Butooro Health Centre [a distant public health facility] to confirm his results just like we instructed them to.”

(VHT Luwala - Farming community)

The providers also worried that HIVST may arouse several **social insecurities** if tested HIV positive especially during unsupervised HIVST. For instance, it may disrupt ART among known HIV positive persons and coercion in case of discordancy in couples which may in turn disrupt would be stable relationships.

“Some…were discordant couples when one does not have HIV and another one has. When one was swallowing tablets not informing the other, we could send him/her for APN [Assisted partner Notification]. APN helps men to see that he informs the wife that he is taking medicine [ART]…we counsel them such that they are able to come to the hospital.”

(Health worker, Nindye Health Centre III)

“The main challenge was among women who delayed to know what happened between them [the VHTs], their husbands and the doctors… They got to know that we keep their husbands’ secrets and wondered why we didn’t let them get involved in the exercise… They could only settle if they let them also get tested.

(VHT Musa – Island)

### Maintenance

This domain focused on understanding program sustainability and the reasons why individual benefits continued or faded, and why the organization delivering the intervention decides to continue or discontinue the intervention – which are important for future program design and scale up [36].

Health providers expressed the need to **optimize tracking of HIVST distribution and use**. They said there was need for a system to account for distributed HIVST, proper reporting of HIVST results, and registration of HIVST entry prior to confirmatory testing and follow-up.

“When a person comes with a used HIVST kit and he gives it to me, I register him again in my book [HIV testing register] because, I will use the kits of the hospital for confirmatory testing. They will be counted and written in books [registered].”

(Health worker, Bunjako Health Centre III)

The providers also mentioned the **need to augment linkage and retention in care** following an HIV-positive self-test. They believed this would further bridge the gap between obtaining a positive HIV diagnosis and progress on the HIV continuum of care.

“He [a certain HIVST user] showed that he does not mind about issues of the medicine [ART]. But later he came back and got some Septrine [Cotrimoxazole tablets]. He came back again and got a package of a month then, he got lost when you have nowhere to look for him again.”

(Health worker, Mitala Maria Health Centre III)

The providers recommended **diversifying HIVST** to include multi-language inserts so as to serve vast populations, and delivery in combined HIV prevention and treatment packs with other products like condoms. They also suggested that subsidizing the cost of HIVST kits to a more affordable price would make them accessible even via private outlets like pharmacies, clinics and drug shops.

“The kit had a multi-language manual and addition, the doctors demonstrated for those who were illiterate… The manuals should be written in known languages.”

(VHT Buwama - Trading Centre)

“… it would be good that where there is a box of condoms there is also one of self-testing kits. It would help a lot…say that as long as there is a condom first test yourself and know your status…”

(Health worker, Mpigi Health Centre IV)

“Others asked why we didn’t have medicine [ARVs] with us to distribute in case someone tested positive and not having to go to hospitals.”

(VHT Buwama – Trading Centre)

The providers recommended expanding and regularly educating the entire community and beyond on HIVST. This includes workplaces, schools, mobile individuals, and health workers who provide HIV testing services. They believed that VHTs could play a crucial role in mobilizing and raising awareness within communities. It is also important for clients to fully comprehend test instructions and for health workers to provide optimal counselling after HIVST.

“I am well known as the VHT among the people and it is my job to always move and let them know of any new program. … There would be testing programs in the area and people would shy away but I was pleased that I mobilized them at my place to use this new method.”

(VHT Nabusanke – Trading Centre)

“We [VHTs] convinced them [the community members] and told them that blood and saliva are both substances from the human body and during these interactions with those that found difficulty, we emphasized going for a blood test in the hospital. I later learnt that this method didn’t reach certain areas and people questioned me on the doctors’ return.”

(VHT Musa – Island)

Health providers also reported that there was need to **differentiate distribution models of HIVST** so as to reach all the last untested persons. They mentioned that there was need to consider controlled but more convenient distribution channels for HIVST such as via local councils, religious leaders and other delivery models utilized for delivery of antiretroviral therapy.

“…it [HIVST] could only be used once and disposed of not knowing where to get another kit as compared to the case of blood tests where one can visit the hospital…these kits should be deployed at hospitals or at the VHTs’ place to be accessible to the people for periodic testing.”

(VHT Nkozi - Trading Centre)

“I got help from religious leaders in churches who gave me a platform and time to address the people.”

(Male, VHT Luwala – Farming community)

The providers emphasized the importance of prioritizing targeted HIVST for at-risk populations such as young people, non-pregnant women, fisher-folk, female sex workers, and other mobile individuals who engage in casual sex.

“Mostly the youth. They are the majority on the landing site and are very sexually active. After a day’s work, most of them engage in sexual activities. I would also recommend it to the elderly men too but mostly people those who live at the landing site.”

(Male, VHT Ggolo – landing site)

“This method targeted only the men but it would have been better if the women were also involved.”

(Male, VHT Musa – Island)

“This method leaves out the blind, mentally unstable, yet it should encompass everyone so that they can have a choice between this and the blood test.”

(VHT Nkozi - Trading Centre)

## Discussion

We explored health provider perspectives of VHT-delivered oral HIVST among men in rural communities of Central Uganda. We found that providers had diverse perspectives of oral HIVST which we summarized using domains of the RE-AIM framework. Perspectives in support of HIVST included being a quick and convenient way to reach men with HIV testing services, a boost for health care seeking, a streamlined and trustworthy approach of HIV testing, able to enhance autonomy about one’s health, well-adapted for community work, and offered opportunity for linkage and re-linkage into care. On the downside, there were concerns about accuracy of HIVST, hesitance to use HIVST, as well as social insecurities and delays in accessing subsequent HIV services after HIVST. Nonetheless, there were recommendations to optimize tracking of HIVST distribution and use, augment linkage and retention in care after HIVST, diversify HIVST for HIV prevention services, broaden sensitization of HIVST users and health providers, and differentiate HIVST kits distribution to suit varied priority populations. Our findings suggest that HIVST can improve men’s involvement and utilization of HIV services but may require further adaptation to optimize gains across the HIV care cascade.

Our study showed that HIVST was a quick and convenient way to reach men with HIV testing services and a boost for health care seeking. This implied that HIVST can be intentionally availed to attract men to seek HIV services irrespective of their location and engagements. These findings agree with prior research conducted among men across varying settings in Uganda and elsewhere in Sub-Saharan Africa which showed high acceptability and uptake of HIVST [2, 9, 41]. These findings strengthen evidence in support of using HIVST to close the remaining gap in testing among laggard populations such as men. Furthermore, this explains why health providers perceived HIVST as a streamlined and trustworthy approach of HIV testing, which can enhance autonomy about one’s health. Nonetheless, uptake of HIVST still encounters challenges in novice populations [8], hence attracting the last underserved persons may require a service that appeals to the target population and can intrinsically trigger their involvement in HIV services without prompting of the health providers [42, 43]. However, there remains a need for continuous sensitization of the health workers to prioritize support systems for clients who seek facility-based services after HIVST.

Our findings further showed that HIVST was well-adapted for community work and offered opportunity for linkage into care and re-linkage (supporting clients who have dropped out of HIV care to resume care). Our findings are consistent with earlier studies that reported high to moderate linkage to care in HIVST among men [10, 11, 13, 44-46]. However, in other perspectives, researchers have suggested that there may not be a single best approach to boosting men’s utilization of HIV linkage services but rather use of combination strategies including home-based ART initiation, use of phone reminders, and community-based ART initiation [15, 42, 43, 47]. Fluctuations in linkage under HIVST have been attributed to several facilitators including communication modalities, navigating of health facility systems and processes, linkage- and psychosocial support, which may be countered by innumerable barriers such as inflexible work schedules, distance to accessing ART, work transfers, denial of HIV-positive results, and fear of stigma and discrimination at health facilities [15]. However, VHTs played a vital role in delivering and facilitating oral HIVST, and through the community-based model can continue to conveniently sensitize and guide communities about HIVST, confirmatory testing and other linkage services such as condom use, ART-initiation and refills, which they may be routinely supporting in their respective communities [44, 48, 49]. There may be need to understand how the facilitators and barriers influencing linkage to care after HIVST can be modified by increasing involvement of VHTs.

Contrary to the perspectives in support of oral HIVST, we also found negative concerns about HIVST including; accuracy, hesitance to the use of HIVST, social insecurities and delays in accessing subsequent HIV services after HIVST. Concerns of accuracy were hinged on the novelty of using oral fluid to test rather than the traditionally accepted blood tests, and were similar to findings from other research which showed expressions of doubt on the accuracy of the self-tests especially for infections that have not lasted for more than 3-months [10, 50-52]. However, in communities where HIVST has been implemented for longer duration, there were diminishing expressions of doubt and hesitance since majority of the population had not only heard of HIVST but had used it and fairly understood the possibility of using oral fluids to test for HIV infection [46, 53]. In most instances, continuity of information flow about HIVST was sustained through peer-to-peer discussions which in turn enhanced health worker-led sensitizations about HIVST conducted at designated kit distribution outlets [46]. Similarly, in settings with broader coverage of HIVST services such as Zambia and Malawi, social insecurities were sporadically reported and minimal to no reports of social or self- harm were found [8, 46]. This further strengthens evidence that the benefits of oral HIVST out-weigh any anticipated or perceived risks of harm [1, 54].

Our study also unveiled suggestions concerning future implementation of oral HIVST including; optimizing tracking of HIVST distribution and use, linkage and retention in care after HIVST as well as diversifying HIVST for HIV prevention services. These findings re-echoe some of the uncertainties surrounding the growing need to embrace HIVST as part of the efforts to close the last 5% gap in HIV testing [47]. Evaluation of HIVST can only be achieved via timely and accurate reporting of issued and used kits, as well as the results obtained. This directly influences appropriateness of programming for the continuously growing population of PLHIV, and HIV prevention services to curb further spread of infection [55]. In addition, upon attaining knowledge of one’s HIV sero-status, timely linkage to services is critical for maintaining a healthy livelihood regardless of one’s sero-status [55-57]. Particularly, timely linkage to ART not only achieves viral suppression among PLHIV, but guarantees longer and healthier lives [58]. In HIVST, linkage to care has been found comparable to that attained via conventional HIV service approaches in the facility and community [14]. However this has been done primarily for short durations of 3 to 6 months after HIVST [3, 14, 59]. There is therefore need for further research to understand the aspects of adherence and retention in care for longer periods beyond one-year of ART following HIVST as well as the other challenges concerning linkage to care in HIVST [60].

Lastly, we found need to broaden and routinize HIVST sensitization among potential users and health providers, as well as differentiate HIVST kits distribution to suit varied priority populations. These results resonate with the desire to extend HIV testing services to underserved sub-groups via HIVST [61]. However, since HIVST is a relatively new approach of HIV testing, there is still need to sensitize the other sub-groups and general population who may not have been prioritized for service initially. Earlier research has shown growing sensitizations among potential users and closely involved health providers [6, 8].

Even then, there remains need to explore and understand strategies for routinizing HIVST sensitization among users and providers. In the same manner, expansion of the HIVST coverage requires diverse approaches of distribution to reach and suit any targeted population [54]. In the recent past several delivery models of HIVST have been studied primarily in isolation[6, 8]. However since there may not be a single best approach to reach all the last untested persons [47], there is still need to explore combination approaches which can be utilized to hasten steps to achieving the 95-95-95 targets.

Our study had limitations. We used transcripts translated from Luganda to English for analysis. Although this was done by experienced members of the study team, there is inherent loss of information during translation [62, 63]. However, we believe its effect may have been minimal since all transcripts were cross-checked for consistency and accuracy and alignment to the study context by the study PI (JN). Secondly, the underlying study targeted men, all data was collected by male research assistants including in that reported in this paper. Therefore, since the study PI was a cis-female, she was not directly involved in data collection but rather relied on interview audio recordings to interpret and present all study findings. The self-description may be need to be considered while utilizing the results. Third, since health workers and VHTs were involved in the parent oral HIVST study for a prolonged period, that could have influenced their perspectives to be more positive. However, we think this may have had a minimal negative effect since the prior involvement equally helped in establishing rapport which was crucial for conduct of this sub-study. Fourth, we categorized emerging themes generated from the data using a framework so may have condensed some information inadvertently. However, since we employed a hybrid inductive-deductive thematic approach to analyze the data, we are confident about the rigor of data and trustworthiness of the findings.

In conclusion, we argue that HIVST boosts testing and subsequent engagement of men in HIV services. Population-wide scale-up of HIVST may require careful consideration of the involvement of health workers, routine sensitization of potential users and health providers, and streamlined tracking HIVST kit distribution, use and reporting of results.

## Abbreviations

ART: Anti-retroviral therapy
COREQ: Consolidated Criteria for Reporting Qualitative Research
HIVST: HIV self-testing
RE-AIM: Reach, Effectiveness, Adoption, Implementation and Adoption
RAs: Research assistants
SSA: Sub-Saharan Africa
VHT: Village Health Team
